# Why don’t they want to donate? Cultural and psychological factors influencing the organ donation intention among Hong Kong University students

**DOI:** 10.1371/journal.pone.0338201

**Published:** 2025-12-19

**Authors:** Wanming Liang, Chi-Shing Tse

**Affiliations:** 1 Department of Educational Psychology, The Chinese University of Hong Kong, Hong Kong, China; 2 Centre for Learning Sciences and Technologies, The Chinese University of Hong Kong, Hong Kong, China; National Research Centre, EGYPT

## Abstract

Despite significant advances in transplantation technologies, the global gap between organ availability and demand continues to widen. Hong Kong, in particular, reports notably low organ donation rates among developed regions. Understanding the psychological barriers to donation is essential for developing effective interventions, particularly among young adults who represent a crucial demographic for establishing lifelong donation commitments. The present study examined the complex interplay of psychosocial and emotional factors influencing organ donation intentions among Hong Kong university students. A cross-sectional survey was conducted with 280 university students in Hong Kong, using validated instruments to assess multiple psychological constructs: depressive thinking, altruism, death anxiety, self-efficacy, perceived social support, Big Five personality traits, and Buddhist and Karmic beliefs. Hierarchical regression and moderated mediation analyses were performed to identify direct and indirect predictors for organ donation intentions. Results showed that higher death anxiety (β = −.133, *p* = .036) and stronger Buddhist beliefs emphasizing bodily integrity (β = −.22, *p* < .001) were associated with lower donation intentions, while greater self-efficacy (β = .22, *p* < .001) and perceived social support (β = .28, *p* < .001) positively predicted donation intentions. Notably, dispositional characteristics including altruism, depressive thinking, personality traits, and Karmic beliefs did not directly predict donation intentions. However, depressive thinking exerted an indirect negative effect on donation intentions through elevated death anxiety, and this mediational pathway was stronger among participants with lower self-efficacy (index of moderated mediation = .035, 95% CI [.0002,.0013]). These findings highlight the critical roles of emotional and situational factors, rather than stable personality characteristics, in determining organ donation intentions among Hong Kong university students. Public health interventions aiming to reduce death anxiety and strengthen self-efficacy may be particularly effective in promoting organ donor registration among young adults in Hong Kong. Future initiatives should prioritize culturally sensitive approaches that address existential concerns while building confidence in donation decision-making.

## Introduction

Organ transplantation remains one of the most transformative medical advances of the twentieth century, offering life-saving treatment for patients with end-stage organ failure [1]. Despite technical progress and rising public awareness, a persistent shortage of donor organs continues to challenge health systems worldwide. In Hong Kong, this problem is particularly acute: as of September 30, 2025, only 409,020 residents out of a population exceeding 7.4 million had registered as potential organ donors, while over 2,000 patients awaited transplants daily [2]. Given that deceased donors constitute the primary source of transplantable organs in Hong Kong, understanding the psychosocial and emotional determinants of posthumous donation intentions is crucial for developing effective interventions to alleviate this shortage.

Prior research has identified multiple psychosocial determinants influencing organ donation intentions, including relational ties, religious beliefs, cultural values, personality traits, altruism, family dynamics, trust in healthcare systems, and demographic factors [3–10]. However, few studies have explicitly investigated these factors within Hong Kong’s unique sociocultural context [11]. To address this crucial research gap, the present study aims to provide a deeper understanding of the psychosocial factors shaping organ donation intentions among Hong Kong university students.

### Psychosocial determinants of organ donation intentions

Previous findings on psychosocial predictors of organ donation intentions, such as knowledge, social atmosphere, personality trait, altruism, self-efficacy, family dynamic, and religious belief, remain inconsistent, particularly within Chinese populations. Among these factors, self-efficacy, defined as the confidence in one’s ability to perform specific behaviors successfully [12], has emerged consistently as a significant predictor of donation intentions [13,14]. For example, Japanese university students who expressed greater self-efficacy regarding their ability to complete organ donation procedures were more likely to register as donors [15]. Table 1 synthesizes the primary areas of contradiction identified in prior research, which form the backdrop for the current investigation.

**Table 1:** Summary of inconsistent findings in prior research on psychosocial predictors of organ donation intentions.

Factor	Commonly Held Belief/ Supporting Finding	Contradictory Evidence from Prior Research	Key Resources
Knowledge about Organ Donation	A higher level of knowledge and understanding about the donation process logically leads to increased willingness to donate.	Multiple studies report finding no significant relationship between the level of donation-related knowledge and the intention to register as a donor.	[16–19]
Family dynamics	Strongly influence donation decisions in collectivistic cultures.	Family views often represent significant barriers or facilitators.	[20–22]
Altruism	As an inherently altruistic act, organ donation is presumed to be strongly and positively predicted by an individual’s general altruistic disposition.	The direct link between altruism and donation intention can become non-significant when other factors, such as social norms or self-efficacy, are statistically controlled.	[6,11,14,23,24]
Personality Traits	Certain personality traits, such as Agreeableness and Conscientiousness, are expected to be reliable predictors of prosocial behaviors like organ donation.	While Agreeableness shows a generally positive correlation, the effect of Conscientiousness is inconsistent across different studies and populations.	[10,25]
Buddhistic & Karmic Beliefs	Beliefs in Karma, which emphasize the consequences of one’s actions, should logically promote prosocial acts like donation to generate positive future outcomes.	The specific relationship between Karmic beliefs and organ donation remains underexplored and unclear, while other religious concerns (e.g., bodily integrity) act as significant barriers.	[1,26–28]

These inconsistencies highlight a critical insight: the decision to donate organs is not the result of a simple, linear calculation. It is a deeply personal and psychologically complex choice influenced by a dynamic interplay of cognitive, emotional, and social factors. A factor that appears decisive in one study may be neutralized or even reversed by another variable in a different context. This underscores the necessity of an integrated approach that examines not just the direct effects of individual factors, but also their interactions.

### Interplay among emotional and psychosocial determinants: self-efficacy, altruism, death anxiety, and depressive thinking

While psychosocial predictors have been extensively studied, emotional determinants such as death anxiety and depressive thinking have received far less attention, particularly in non-Western populations. Death anxiety, refers to negative emotional responses specifically triggered by mortality awareness [14], may influence organ donation in complex ways. According to Terror Management Theory [29], individuals employ psychological defenses to manage existential anxieties. Such defense involves engaging in prosocial behavior like organ donation to achieve symbolic immortality strategies or cultural worldview affirmations. In other words, heightened death anxiety may motivate altruistic actions, such as organ donation, which can serve as a buffer against the discomfort associated with mortality awareness [30]. However, empirical findings remain inconclusive, with studies reporting both negative associations (heightened death anxiety reduces willingness to donate due to avoidance of death-related topics, e.g., [31–33] and no significant relationship [34]. Furthermore, self-efficacy may moderate death anxiety effects. Jessop and Wade [35] indicates that individuals with higher self-efficacy are less susceptible to the negative effects of death anxiety, whereas those with lower self-efficacy were more likely to avoid action. Given the potential moderating role of self-efficacy, the present study examines how self-efficacy interacts with death anxiety to shape organ donation intentions among Hong Kong university students, an area warranting deeper empirical investigation.

Depressive thinking, characterized by persistent feelings of sadness, dissatisfaction, or self-critical thoughts, often precedes clinical depression [36]. This study specifically targets young adults who occasionally experience such thoughts but do not meet clinical criteria for depressive disorder. Depressive states often correlate with reduced altruistic engagement and prosocial behaviors [37,38], with helping others promoting self-healing of depression [38]. However, recent studies suggest that depressive experiences may stimulate empathy and altruistic responses via processes akin to posttraumatic growth or “altruism born of suffering” [39]. An illustrative social media post, “I don’t deserve a good life, but others do” [40], reflects how depressive cognition may prompt altruistic behaviors like organ donation. Given these mixed evidence, clarifying how depressive thinking interacts with altruism and death anxiety to influence organ donation intentions is essential.

### The present study

Although prior research has examined these psychosocial and emotional determinants separately, their unique and interactive influences remain poorly understood. To address this gap, the present study investigates the interplay among psychosocial and emotional variables influencing organ donation intentions in Hong Kong university students (aged 18–25), a demographic previously identified as more positively disposed toward organ donation compared to older age groups (aged 31–70) in Chinese contexts [1,41].

The Theory of Planned Behavior [42] and Theory of Reasoned Action [43] provide robust frameworks for investigating organ donation behaviors, positioning individual intentions as key predictors of donation behaviors, influenced by attitudes, subjective norms, and perceived behavioral control [44]. Guided by these frameworks, the current study explores the predictive roles of demographic, psychosocial, and emotional variables in predicting organ donation intentions among Hong Kong university students. Demographic factors, including age, gender, educational background, family socioeconomic status, and personal income, were considered, as they were found to be associated with donation willingness [10,45,46].

Specifically, we address two overarching research questions

*RQ1*: Do psychosocial and emotional factors (knowledge, social atmosphere, personality traits, self-efficacy, altruism, family dynamics, Buddhism/Karma beliefs, depressive thinking, death anxiety) uniquely predict organ donation intentions after controlling for other variables?

*RQ2*: How do depressive thinking, altruism, death anxiety, and self-efficacy interact to influence organ donation intentions?

Based on these questions, we derive 15 hypotheses. We expect that organ donation intentions will be higher for those with greater knowledge about organ donation (H1), stronger perceived social support from family and friends (H2), greater social media usage (H3), higher conscientiousness (H4), higher agreeableness (H5), elevated self-efficacy (H6), increased altruism (H7), stronger Buddhist beliefs (H8), stronger Karmic beliefs (H9), healthier family dynamics (H10), higher depressive thinking (H11), and lower death anxiety (H12). In addition to these direct relationships, we propose three hypotheses examining moderation and mediation effects (see supporting information - S1 Appendix or in https://osf.io/ajb65/files/dpax9 for conceptual models). First, altruism is expected to moderate the positive relationship between self-efficacy and organ donation intentions, such that this association will be stronger for highly altruistic individuals (H13). Second, altruism and death anxiety are expected to mediate the relationship between depressive thinking and organ donation intentions (H14). Depressive thinking may enhance altruism, thereby increasing donation intentions, or it may heighten death anxiety, thereby reducing donation intentions. Third, we propose a moderated mediation model wherein self-efficacy moderates the indirect effects of depressive thinking on organ donation intentions through altruism and death anxiety (H15). These indirect pathways would vary according to levels of self-efficacy.

Overall, the present study aims to provide a more integrated understanding of how psychosocial and emotional variables jointly shape posthumous organ donation intentions among Hong Kong university students. While prior research on this population has been limited, with one notable study focusing primarily on bodily integrity concerns [47], there remains a lack of updated, multifaceted investigations that consider a broader range of integrated psychosocial and emotional predictors. By addressing this gap, the present study offers timely insights into the complex determinants underlying organ donation intentions among a critical yet understudied demographic, ultimately informing targeted health communication strategies and evidence-based interventions in Hong Kong.

## Method

### Participants

A total of 280 undergraduate students (74.5% female; *M*ₐ₉ₑ = 20.00, *SD* = 1.00) were recruited from the Chinese University of Hong Kong between February and April 2025. Although relying solely on university students may limit generalizability to wider young adult populations, this sample provides a relevant starting point for examining organ donation intentions in detail.

A priori power analysis using G*Power [61] indicated a required sample size of 263 to detect a medium effect (*f² = *0.15) [48] in a multiple regression model (fixed model, R² increase), with 16 predictors (knowledge, two components of social atmosphere, five personality traits, altruism, self-efficacy, family dynamics, beliefs in Buddhism and Karma, depressive thinking, death anxiety, and the interaction between self-efficacy and altruism), 6 covariates (gender, age, education, individual monthly income, family monthly income, parental education), α = .05, and power = .99. To account for potential attrition or incomplete data, the target sample was increased to 280 participants. Participants received monetary compensation (120 HKD) for their participation.

The study received ethical approval from the Survey and Behavioural Research Ethics Committee of the Chinese University of Hong Kong (Ref. No. EDU2025−034). Written informed consent was obtained electronically before participation. A pilot study (N = 20) was conducted to assess questionnaire comprehensibility, and feedback informed minor adjustments to item phrasing and response formats.

### Materials and procedure

Given our focus on depressive thinking, as referred to as negative thought patterns distinct from clinical depression, participants reporting a prior clinical depression diagnosis were excluded.

Data were collected via an online questionnaire provided bilingually (Chinese and English). Established measures adapted from prior research included the *Family Health Questions and Assignment Criteria* [49], *Big Five Inventory (BFI-44)* [50], and *Automatic Thoughts Questionnaire – Negative (ATQ-N)* [51].

Other scales underwent rigorous translation and back-translation procedures. Specifically, two bilingual translators independently translated original English items into Chinese. Researchers reviewed these translations to produce a consensus version, and subsequently back-translated into English by two additional bilingual speakers unaware of the original items. Final translations were carefully reviewed for semantic equivalence and cultural appropriateness (full questionnaire available in Supporting information - S2 Appendix, https://osf.io/ajb65/files/6mtk2). Further refinements were implemented based on pilot study findings.

The instrument also included demographic items (age, gender, education level, individual and family monthly income, parental education levels, and previous clinical diagnosis of depression) and measures assessing organ donation intentions and registration status, adapted from Chen et al. [1].

*Organ donation decision-making* was assessed by two items adapted from established measures [1,10,52–54]: (a) intention (“*When your life cannot be saved, are you willing to donate organs?”*), rated on a 7-point Likert scale (1 = extremely unlikely, 7 = extremely likely); and (b) registration status (“*Have you ever registered for organ donation?*”), with a binary response (0 = No, 1 = Yes).

Psychosocial and emotional factors were measured as follows:

- Organ Donation Knowledge: Assessed via a 10-item true-or-false scale. Correct responses were summed, yielding scores from 0 to 10, with higher scores indicating greater knowledge (α = .74, [54].
- Social Atmosphere: Included two components: (a) Social interaction/support (6 items adapted from D’Alessandro et al. (8; α = .77), measuring perceived social support and interactions regarding organ donation on a 5-point Likert scale (1 = strongly disagree; 5 = strongly agree), with higher scores indicating greater social interaction/support of organ donation; and (b) Social media usage (items adapted from Gong et al. [55]; α = .70), assessing frequency of social media exposure using a 5-point scale (1 = never, 5 = almost every day). Participants also reported the number of days they were exposed to various media in a week (0–7). Scores were summed, with higher totals indicating greater media usage.
- Personality Traits: Evaluated with the 44-item *Big Five Inventory (BFI)*, assessing neuroticism, extraversion, openness to experience, agreeableness, and conscientiousness [56,57] on a 5-point Likert scale (α = .70−.80) [50].
- Self-efficacy: Three items adapted from Brug et al. [58] assessed perceived ease of organ donor registration, rated on a 5-point Likert scale. Responses were summed, with higher scores denoting greater self-efficacy (α = .87; [11].
- Family Dynamics: Nine items adapted from *Family Health Questions and Assignment Criteria* measured perceived family relationship quality [10,49], using a 5-point Likert scale Higher scores indicated stronger perceived familial trust and connectedness (α = .83; [10].
- Beliefs in Buddhism: 3 items adapted from Lam and McCullough’s [59] *Belief in Buddhism* scale was used. Given noted contradictions regarding organ donation within Buddhist teachings, all items were included to capture overall religious beliefs. Items were rated on a 5-point Likert scale with higher scores indicating stronger beliefs in Buddhism.
- Beliefs in Karma [Beliefs in Buddhism and Karma were retained separately because they represent distinct cognitive–spiritual orientations: Buddhism beliefs emphasize bodily integrity and postmortem peace, whereas Karmic beliefs focus on moral causation and reciprocity. Although related, both have unique implications for donation willingness in East Asian contexts.]: Beliefs were measured using 16 items from White et al.’s [60]

*Belief in Karma Questionnaire*, with higher scores indicating stronger beliefs in Karma (α = .94).

- Altruism: Eleven items adapted from Morgan and Miller [61] assessed altruistic attitudes. Negatively phrased items were reverse-scored. Items were rated on a 5-point Likert scale with higher summed scores indicating greater altruism (α = .84; [62]).
- Depressive Thinking: Measured by the 30-item *Automatic Thoughts Questionnaire – Negative (ATQ-N)* [36] that assesses frequency of automatic negative thoughts characteristic of depressive thinking [51], capturing habitual negative cognitions distinct from clinical depression. Items were rated on a 5-point frequency scale (1 = not at all; 2 = sometimes; 3 = moderately often; 4 = often; 5 = all the time), with higher summed scores indicating more frequent depressive thinking (α = .95).
- Death Anxiety: Evaluated using the 17-item *Death Anxiety Inventory (DAI)* [14] rated on a 5-point Likert scale. Higher summed scores indicated greater death anxiety (α = .89).

### Data analyses

All analyses were conducted using IBM SPSS Statistics (Version 29) with the PROCESS macro (Version 4.2) [63]. Prior to analysis, assumptions of normality, linearity, and multicollinearity were examined. Descriptive statistics and bivariate correlations were computed for all study variables.

To assess the relative contribution of key predictors to organ donation intention, hierarchical multiple regression analyses were performed. Demographic variables (e.g., age, gender, religious) were entered in Step 1, followed by psychological and emotional variables (depressive thinking, death anxiety, altruism, self-efficacy, and Buddhist/Karmic beliefs) in Step 2.

To further test the hypothesized relationships among variables, a series of mediation and moderation analyses were performed using the PROCESS macro. A separate moderation analysis (Model 1) examined whether altruism moderated the relationship between self-efficacy and donation intention, controlling for death anxiety, Buddhist beliefs, and perceived social interaction/support. Model 4 was used to examine whether death anxiety and altruism mediated the effect of depressive thinking on organ donation intention. Model 14 was applied to test a moderated mediation model, in which self-efficacy moderated the indirect effects of depressive thinking on organ donation intention via death anxiety and altruism.

All continuous variables were standardized before computing interaction terms. Bootstrapping with 5,000 resamples was used to estimate 95% bias-corrected confidence intervals for indirect and interaction effects, which were considered statistically significant if zero was not included in the confidence interval.

## Results

### Demographic characteristics

All data, code, and materials are available at https://osf.io/ajb65/. The majority reported family monthly household incomes between 10,000 and 49,999 HKD, with varied parental education levels. Over 80% reported individual monthly incomes below 4,000 HKD (see Table 2 for detailed demographic characteristics).

### Predictors of Organ Donation Intentions (H1-H12)

*Organ Donation Intentions (Q1)*. To identify psychosocial and emotional predictors of organ donation intentions, we conducted hierarchical regression analyses with standardized (z-scored) variables.

Step 1(demographic covariates: gender, age, family income, parental education, and personal income) did not significantly predict donation intention, *R²* = .010, *F* (6,273)=.437, *p* = .854. In Step 2, adding psychosocial and emotional predictors significantly improved the model fit, *R²* = .370, *F* (21,258)=7.216, *p* < .001, indicating substantial explanatory power beyond demographics. Multicollinearity was assessed using variance inflation factor (VIF) values. All VIF values in the final model ranged from 1.071 to 1.959, indicating no significant multicollinearity concern.

In this model, social interaction/support (*B* = .10, *SE* = .02, β = .28, *p* < .001) and self-efficacy (*B* = .12, *SE* = .03, β = .22, *p* < .001) emerged as strong positive predictors of donation intention, supporting H2 and H6. In contrast, Buddhist beliefs toward organ donation (*B* = −.14, *SE* = .04, β = −.22, *p* = .002) and death anxiety (*B* = −.015, *SE* = .007, β = −.133, *p* = .033) were significant negative predictors, supporting H12. Narratively, these findings suggest that individuals with higher perceived social support and self-efficacy are more willing to donate organs, whereas stronger Buddhist beliefs and higher death anxiety are associated with lower willingness to donate. Correlation and regression coefficients are presented in Tables 3 and 4.

**Table 2:** Participants’ demographic variables.

		Count	N %
Gender	Male	66	23.6%
	Female	214	76.4%
Grade Level	Year 1	67	23.9%
	Year 2	59	21.1%
	Year 3	62	22.1%
	Year 4	70	25.0%
	Year 5	22	7.9%
Father’s education level	Lower secondary or below	91	32.5%
	Higher secondary/ diploma	119	42.5%
	Bachelor’s degree	47	16.8%
	Postgraduate degree or above	23	8.2%
Mother’s education level	Lower secondary or below	103	36.8%
	Higher secondary/ diploma	128	45.7%
	Bachelor’s degree	39	13.9%
	Postgraduate degree or above	10	3.6%
Family household income	<10,000 HKD	18	6.4%
	10,000–29,999 HKD	106	37.9%
	30,000–49,999 HKD	83	29.6%
	50,000–69,999 HKD	39	13.9%
	70,000–89,999 HKD	17	6.1%
	>=90,000 HKD	17	6.1%
Individual monthly income	<1,000 HKD	89	31.8%
	1,000–3,999 HKD	138	49.3%
	4,000–5,999 HKD	40	14.3%
	6,000–7,999 HKD	1	0.4%
	8,000–9,999 HKD	7	2.5%
	>=10,000 HKD	5	1.8%

**Table 3:** Pearson correlation among variables (N = 280).

	*M*	*SD*	1	2	3	4	5	6	7	8	9	10	11	12	13	14	15	16	17
1.Donation Intention	5.58	0.36	--																
2.KlgOD	7.56	1.28	0.11	--															
3.SocSup	18.21	3.95	0.43^**^	0.09	--														
4.SocTyp	--	--	0.09	0.04	0.04	--													
5.SocUse	10.52	1.46	0.17^**^	0.14^*^	0.09	0.25^**^	--												
6.Ext	25.61	3.16	0.05	0.02	0.23^**^	−0.08	0.16^**^	--											
7.Agr	27.91	3.49	0.13^*^	0.04	0.20^**^	−0.07	.014^*^	−.40^**^	--										
8.Con	30.35	3.20	−0.01	0.13^*^	0.10	−0.11	0.15^*^	0.44^**^	0.31^**^	--									
9.Neu	26.28	3.08	−0.04	−0.05	−0.07	−.038	0.09	.037	0.28^**^	0.30^**^	--								
10.Ope	30.05	5.21	0.02	−0.04	0.18^**^	−.045	−0.01	0.42^**^	0.20^**^	0.25^**^	0.14^*^	--							
11.SelEff	7.06	2.75	0.44^**^	0.13^*^	0.35^**^	.109	0.08	−0.07	−0.01	−0.04	−0.14^*^	−0.12^*^	--						
12.FamDyn	32.42	5.30	0.15^*^	0.05	0.29^**^	.006	0.18^**^	0.25^**^	0.11	0.12^*^	−0.12^*^	0.21^**^	0.16^**^	--					
13.BudOD	6.54	2.14	−0.29^**^	−0.19^**^	−0.08	−0.15^*^	−0.13^*^	0.07	0.09	0.11	0.15^**^	0.15^*^	−.025^**^	0.02	--				
14.BelKar	42.28	12.23	−0.05	−0.12^*^	0.06	−0.09	−0.08	0.08	.070	0.11	0.09	0.07	−.015	.083	0.58^**^	--			
15.Altru	36.09	5.86	0.23^**^	−0.01	0.33^**^	−0.01	0.13^*^	0.49^**^	0.36^**^	0.32^**^	0.04	0.29^**^	0.18^**^	0.41^**^	0.03	0.15^*^	--		
16.DepThi	77.80	24.89	−0.03	0.01	−0.09	−0.10	−0.12	−0.31^**^	0.04	−0.05	0.31^**^	−.175^**^	−0.06	−0.42^**^	.044	−0.00	−0.29^**^	--	
17.DeaAnx	41.19	12.43	−0.32^**^	−0.05	−0.20^**^	−0.08	−0.13^*^	0.04	0.12^*^	0.11	0.34^**^	0.1	−0.36^**^	−0.13^*^	0.38^**^	0.31^**^	−0.04	0.19^**^	--

Note. KlgOD = Knowledge of Organ Donation SocSup = Social interactional/support; SocTyp = Social media type; SocUse = Social media use; Ext = Extraversion; Agr = Agreeableness; Con = Consciousness; Neu = Neuroticism; Ope = Openness; SelEff = Self-efficacy; FamDyn = Family dynamics; BudOD = Buddhist beliefs toward organ donation; BelKar = Kamric beliefs; Altru = Altruism; DepThi = Depressive thinking; DeaAnx = Death anxiety; Correlation is significant at the 0.01 level (2-tailed). *p < .05. **p < .01. ***p < .001

**Table 4:** Results of hierarchical regression analysis on psychological and emotional factors influencing organ donation intentions.

Model		Standardized Coefficients β	*t*	F Change	*R²* Change
1	(Constant)		5.38***	0.44	0.01
	Gender	−0.04	−0.73		
	Age	−0.05	−0.75		
	Family household income	0.01	0.12		
	Father’s education level	−0.02	−0.28		
	Mother’s education level	0.02	0.23		
	Individual monthly income	−0.06	−1.00		
2	(Constant)		6.54***	9.84***	0.36***
	Gender	−0.08	−1.54		
	Age	−0.04	−0.71		
	Family household income	0.011	0.19		
	Father’s education level	−0.03	−0.43		
	Mother’s education level	−0.03	−0.48		
	Individual monthly income	−0.07	−1.25		
	Organ donation knowledge	0.01	0.19		
	**Social interaction/support**	**0.28**	4.83***		
	Social media use	0.11	1.944		
	Extraversion	−0.05	−0.69		
	Agreeableness	0.06	0.96		
	Consciousness	−0.07	−1.07		
	Neuroticism	0.07	1.14		
	Openness	0.03	0.60		
	**Self-efficacy**	**0.23**	3.80***		
	Family dynamics	−0.01	−0.12		
	**Buddhist beliefs toward organ donation**	**−0.22**	−3.20**		
	Karmic beliefs	0.11	1.72		
	Altruism	0.09	1.38		
	Depressive thinking	0.04	0.62		
	**Death anxiety**	**−0.13**	−2.15*		

Note. *p < .05; **p < .01; ***p < .001

*Organ Donation Registration Status (Q2).* Only 7.9% of participants (22 of 258) reported being registered organ donors. Due to the small number of registered donors, logistic regression analyses failed to converge. Thus, subsequent analyses focused exclusively on **donation intentions** rather than actual registration status.

### Moderation analysis: altruism, self-efficacy, and organ donation intention (H13)

A moderation analysis PROCESS Model 1 examined whether altruism moderated the relationship between self-efficacy and donation intention, controlling for death anxiety, Buddhist beliefs, and social interaction/support.

The overall model was significant, *F*(6,273)=22.86, *p* < .001, *R²* = .33. However, the self-efficacy × altruism interaction was nonsignificant (*B* = 0.06, *SE* = 0.05, *t* = 1.29, *p* = .20, 95% CI [−0.03, 0.16]; ΔR² = .004, *F*(1,273)=1.67, *p* = .20), indica*t*ing that altruism did not moderate the relationship between self-efficacy and organ donation intention. Thus, H13 was not supported.

### Mediation analysis: roles of altruism and death anxiety in linking depressive thinking and donation intentions (H14)

Parallel mediation analyses (PROCESS Model 4; 5,000 bootstrap samples) [63] tested whether altruism and death anxiety mediated the relationship between depressive thinking and donation intention, controlling for self-efficacy, Buddhist beliefs toward organ donation, and social interaction/support.

Results showed that depressive thinking predicted lower altruism (*B* = −.26, *SE* = .05, *t* = −4.83, *p* < .001, β = −.26), and higher death anxiety (*B* = .16, *SE* = .05, t = 3.00, *p* = .003, β = .16). In turn, altruism positively predicted donation intention (*B* = .11, *SE* = .05, *t* = 2.06, *p* = .005 β = .11), while dea*t*h anxiety negatively predicted donation intention (*B* = −.12, *SE* = .06, *t* = −2.11, *p* = .036, β = −.12).

The direct effect of depressive thinking on donation intentions was nonsignificant, *B* = .07, *SE* = .05, *t* = 1.30, *p* = .192, 95% CI [−.035,.172], β = .07. The total indirect effect through altruism and death anxiety combined was significant (*effect* = −.049, BootSE = .022, 95% CI [−.096, −.001]). However, when examined individually, neither indirect pathway alone reached significance (altruism effect = −.020, BootSE = .019, 95% CI [−.070,.003]; death anxiety effect = −.020, BootSE = .013, 95% CI [−.048,.001].

Overall, the results suggest that depressive thinking indirectly reduced organ donation intention primarily through increasing death anxiety and reducing altruistic motivation, although the direct path from depressive thinking to donation intention was nonsignificant.

### Moderated mediation analysis: self-efficacy moderating indirect effects of depressive thinking (H15)

A moderated mediation analyses (PROCESS Model 14) was tested to examine whether self-efficacy moderated the indirect paths linking depressive thinking to donation intentions via altruism and death anxiety, controlling for social interaction/support and Buddhist beliefs toward organ donation. The overall model significantly predicted donation intention, *F*(8,271)=21.94, *p* < .001, *R²* = .39.

Depressive thinking significantly predicted lower altruism (*B* = −.27, *SE* = .05, *t* = −4.86, *p* < .001) and higher death anxiety (*B* = .16, *SE* = .05, *t* = 3.02, *p* = .003), bu*t* did not directly predict donation intentions (*B* = .07, *SE* = .05, *t* = 1.30, *p* = .194). Al*t*ruism positively predicted donation intentions (*B* = .13, *SE* = .05, *t* = 2.29, *p* = .041), while dea*t*h anxiety did not directly predict intention in this moderated model (*B* = −.07, *SE* = .06, *t* = −1.16, *p* = .247).

Self-efficacy directly predicted intention (*B* = .23, *SE* = .05, *t* = 4.27, *p* < .001). Importantly, the death anxiety × self-efficacy interaction was significant (*B* = .22, *SE* = .04, *t* = 4.95, *p* < .001, 95% CI [.130,.302]), con*t*ributing a significant incremental variance (*ΔR²* = .055, *F*(1,271)=24.55, *p* < .001). In contrast, the altruism × self-efficacy interaction was not significant (*p* = .116). This suggests that the moderating effect of self-efficacy was specific to the pathway involving death anxiety.

**Fig 1:**
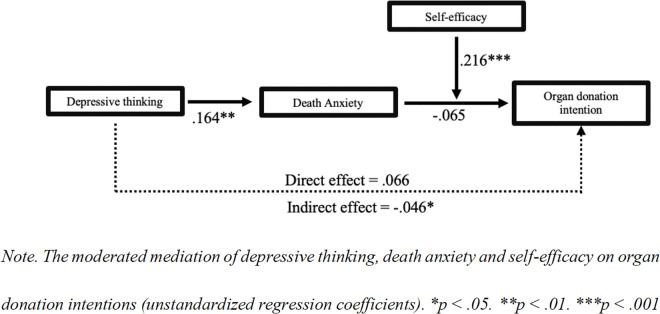
Moderated mediation analysis of depressive thinking on organ donation intentions through death anxiety, moderated by self-efficacy.

**Fig 2:**
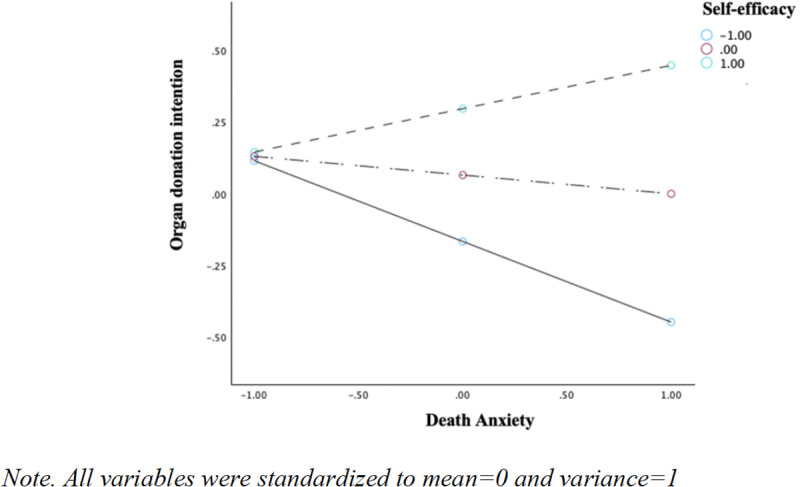
Self-efficacy as a moderator between death anxiety and organ donation intentions.

Probing the significant death anxiety × self-efficacy interaction showed that the indirect effect via death anxiety was significant only at low level of self-efficacy (−1*SD*, *Effect* = −.046, 95% CI [−.0911, −.0118]), but not at mean or high level (+1*SD*). Thus, self-efficacy serves as a buffer against the detrimental impact of death anxiety on donation intentions. The index of moderated mediation was significant for death anxiety, *Index* = .035, 95% CI [.011,.065], confirming moderation by self-efficacy only along the death anxiety pathway and partially supporting H15 (see Figs 1–2). No moderation was observed via altruism (*Index* = −0.02, 95% CI [−.057,.017]).

In sum, our findings highlight death anxiety and self-efficacy as critical factors shaping organ donation intentions among Hong Kong university students. Depressive thinking reduces willingness to donate indirectly by elevating death anxiety, while high self-efficacy mitigates this adverse effect. These results underscore the importance of strengthening individual self-efficacy to counteract existential anxieties and promote prosocial health behaviors.

## Discussion

### Summary of major findings

The present study examined psychosocial and emotional determinants of organ donation intentions among Hong Kong university students, emphasizing the mediating roles of altruism and death anxiety, and the moderating role of self-efficacy. Aligning with Morgan and Miller’s [61,64] organ donation framework, our findings indicate that organ donation intentions are not solely rational decisions but are substantially influenced by emotional factors, particularly death anxiety. Heightened death anxiety was associated with reduced intentions to donate organs [32,65]. Self-efficacy emerged as a robust positive predictor, both directly increasing intentions and buffering against the negative effect of death anxiety. Social support also significantly predicted higher donation intentions, reflecting the importance of interpersonal validation in Hong Kong’s relational-oriented culture. In contrast, altruism and knowledge did not directly predict intentions, suggesting that prosocial disposition or factual understanding alone may be insufficient to overcome existential and cultural barriers.

### Interpretation using established frameworks

Drawing on Terror Management Theory [49], contemplation of organ donation may activate existential fears, eliciting avoidance responses that reduce willingness to donate. Cultural beliefs regarding bodily integrity and reincarnation, particularly within Buddhist frameworks, further influence donation intentions. While younger cohorts in Hong Kong show increasing acceptance of organ donation, our findings indicate that culturally ingrained concerns about bodily completeness remain salient, consistent with prior work on Chinese populations [47]. Our results also revealed a negative relationship between Buddhist beliefs emphasizing bodily integrity and organ donation intentions. The measure explicitly highlighted beliefs regarding maintaining bodily intactness after death and implications for reincarnation (beliefs about the next life). While prior work indicated a gradual shift toward greater acceptance of organ donation in Hong Kong and traditional beliefs regarding bodily integrity were gradually waning [66], our findings suggest that culturally ingrained beliefs about bodily integrity and reincarnation persist among younger cohorts, particularly regarding afterlife concerns [66–68]. Similar concerns regarding reincarnation and bodily completeness were observed among Chinese American populations [69], underscoring cross-cultural consistencies in this barrier.

Corroborating previous findings [6,10], perceived social support significantly associates with greater donation intentions. In Hong Kong, a society where interpersonal relationships and social harmony are highly valued, endorsement from family and peers likely provides essential validation, facilitating openness toward donation. These findings highlight the importance of fostering supportive interpersonal environments and open dialogue as integral components of organ donation promotion strategies [70,71].

Contrary to our hypothesis and previous findings [14,61,72], altruism neither directly predicted donation intentions nor moderated the relationships examined. This unexpected result challenges the view that prosocial dispositions reliably translate into willingness to donate, particularly in contexts involving existential concerns or bodily interventions. Nevertheless, our findings align with emerging perspectives suggesting altruistic traits alone may be insufficient to motivate donation intentions when emotional barriers such as death anxiety are salient [73,74]. Similarly, factual knowledge about organ donation did not predict donation intention, despite generally high knowledge levels in our sample (*M* = 7.56 out of 10). This finding diverges from earlier studies linking increased knowledge to greater willingness [6]. The limited variance (SD = 1.28) in knowledge scores among our educated sample likely attenuated its predictive power. Importantly, this highlights the persistent knowledge-action gap, suggesting that informational interventions alone may inadequately address underlying emotional and existential barriers, such as death anxiety.

Further, personality traits measured by the Big Five Inventory were unrelated to donation intentions, contrasting with prior research identifying agreeableness and conscientiousness as significant predictors [75]. Similarly, while Buddhist beliefs significantly predicted donation intention, karmic beliefs showed limited effects. These observations suggest situational and emotional factors may overshadow dispositional personality traits and general religious worldviews in predicting donation intentions within this demographic and cultural context. Hence, intervention strategies emphasizing context-specific psychological determinants, particularly self-efficacy and social support, may be more effective than those focused exclusively on stable personality or broad religious beliefs.

Depressive thinking indirectly reduced intentions via heightened death anxiety and diminished altruism, neither pathway alone reached full statistical significance. However, the indirect effect through death anxiety approached significance, corroborating prior evidence suggesting depressive cognition may exacerbate existential fears, thereby inhibiting prosocial action [76]. These findings underscore the complexity of emotional responses to mortality in decisions about death-related prosocial behaviors. Supporting this idea, Blackie and Cozzolino [77] found that mortality salience could enhance prosocial intentions, such as blood donation, yet noted this effect diminishes with avoidance tendencies common in depressive states. However, Zeng and Tse [78] argued that stable cultural orientations and relational self-esteem more strongly moderate mortality salience effects on prosocial behaviors than immediate emotional reactions. Xiao et al. [79] found that Chinese participants did not show a reduced willingness to donate organs under mortality salience, diverging from findings in Western samples where avoidance and defensive reactions to death reminders often lead to lower prosocial intentions [80]. Together, these mixed findings highlight the need for deeper exploration of how stable traits and cultural worldviews interact with existential emotions to influence organ donation intentions.

Self-efficacy emerged as a robust positive predictor of donation intention, affirming its critical role in facilitating health-related behaviors [14]. Increased self-efficacy may enhance individuals’ perceptions of agency and control over end-of-life decisions, thereby mitigating existential anxieties and encouraging a proactive approach to organ donation. Indeed, higher self-efficacy was associated with lower death anxiety, *r* = −.36, *p* < .001, reflecting a sense of personal empowerment and reduced vulnerability towards existential concerns [81]. More importantly, self-efficacy significantly moderated the negative impact between death anxiety and donation intention: individuals with high self-efficacy appeared buffered against the detrimental impact of death anxiety, highlighting its protective role.

### Public health and practical implications

Our results emphasize several modifiable factors that can inform intervention strategies. First, enhancing self-efficacy appears critical. Individuals with higher self-efficacy are more confident in their ability to make informed decisions about organ donation and are less influenced by existential anxieties. Intervention programs could include skills-building workshops that educate students about organ donation procedures, clarify misconceptions, and guide participants through decision-making exercises. For example, interactive simulations or decision aids could help individuals practice making donation-related choices, thereby strengthening their sense of control and personal agency. Moreover, campaigns could emphasize stories of peers successfully registering as donors to model positive behaviors and reinforce efficacy.

Second, fostering supportive social environments is essential, especially in culturally relational contexts like Hong Kong where family and peer opinions carry considerable weight [82]. Programs could encourage family discussions about organ donation, incorporating culturally sensitive materials that respect beliefs about bodily integrity and afterlife concerns while providing clear explanations of donation processes. Peer-led initiatives and mentorship programs could further normalize donation as a prosocial behavior, taking advantage of the influence of social networks to create a sense of collective endorsement. Public health campaigns could combine these approaches, integrating informational content, emotional support strategies, and social endorsement, to reduce fear, mitigate existential anxiety, and increase willingness to donate. By targeting these modifiable psychosocial factors, interventions can move beyond simple knowledge dissemination toward empowering individuals and creating socially supportive contexts that facilitate actual registration and donation behaviors.

### Limitations and future directions

Despite its contributions, this study has limitations. First, its cross-sectional design precludes causal inferences. Future longitudinal or experimental studies are necessary to determine whether reducing death anxiety or increasing self-efficacy causally enhances organ donation intentions and actual registration behaviors. Second, reliance on self-report measures introduces potential biases, such as social desirability or recall inaccuracies. Future research could integrate behavioral tasks, implicit measures, or experimental manipulations to validate and extend present findings. Third, our mediation and moderation analyses focused primarily on depressive thinking, death anxiety, altruism, and self-efficacy. Future research should explore other potential mediators (e.g., empathy, relational self-esteem) linking personality traits to donation intentions [75]. Finally, the substantial intention-action gap warrants further investigation into psychological, social, and contextual factors hindering translation of positive intentions into donor registration behaviors.

Importantly, these limitations also present opportunities for applied interventions and policy initiatives. For instance, public health campaigns or university-based donation drives could specifically aim to reduce death anxiety, enhance self-efficacy, and leverage peer and family support to facilitate donor registration. Cross-cultural comparisons with regions exhibiting higher donation rates (e.g., Spain or the United States) could further identify systemic and cultural facilitators, such as opt-out policies, that might be adapted to the Hong Kong context. In conclusion, addressing these limitations offers a roadmap for translating theoretical insights into practical strategies that increase organ donation rates.

## Supporting information

S1 Appendix Conceptual models of the study. (PDF)

S2 Appendix Full questionnaire of the study. (PDF)

## References

[1] Chen X, Wei W, Ai W. Organ donation: Key factors influencing the younger generation’s decision-making in China. Front Public Health. 2023;11:1052875. doi: 10.3389/fpubh.2023.1052875 36815163 PMC9940821

[2] Chu ST-W, Chung PPW, Hui YL, Choi HC, Lam HW, Sin LL, et al. Knowledge and attitude regarding organ donation among medical students in Hong Kong: a cross-sectional study. Postgrad Med J. 2023;99(1173):744–52. doi: 10.1136/pmj-2022-141781 37117044

[3] Ahmadian S, Khaghanizadeh M, Khaleghi E, Hossein Zarghami M, Ebadi A. Stressors experienced by the family members of brain-dead people during the process of organ donation: A qualitative study. Death Stud. 2020;44(12):759–70. doi: 10.1080/07481187.2019.1609137 31058581

[4] Birtan D, Arslantas MK, Dincer PC, Altun GT, Bilgili B, Ucar FB, et al. Effect of Interviews Done by Intensive Care Physicians on Organ Donation. Transplant Proc. 2017;49(3):396–8. doi: 10.1016/j.transproceed.2017.01.030 28340798

[5] Castillo-Angeles M, Li G, Bain PA, Stinebring J, Salim A, Adler JT. Systematic review of hospital-level metrics and interventions to increase deceased organ donation. Transplant Rev (Orlando). 2021;35(3):100613. doi: 10.1016/j.trre.2021.100613 33744820

[6] D’Alessandro AM, Peltier JW, Dahl AJ. The impact of social, cognitive and attitudinal dimensions on college students’ support for organ donation. Am J Transplant. 2012;12(1):152–61. doi: 10.1111/j.1600-6143.2011.03783.x 21992480

[7] Irving MJ, Tong A, Jan S, Cass A, Rose J, Chadban S, et al. Factors that influence the decision to be an organ donor: a systematic review of the qualitative literature. Nephrol Dial Transplant. 2012;27(6):2526–33. doi: 10.1093/ndt/gfr683 22193049

[8] Ruta F, Gallo G, Ferrara P, Terzoni S, Monica AD, Dal Mas F, et al. Translating Knowledge About Organ and Tissue Donation Using Webinars: An Exploratory Study In Italy. Transplant Proc. 2021;53(6):1792–7. doi: 10.1016/j.transproceed.2021.06.018 34275598

[9] Zhang Q-X, Xie J-F, Zhou J-D, Xiao S-S, Liu A-Z, Hu G-Q, et al. Impact Factors and Attitudes Toward Organ Donation Among Transplantation Patients and Their Caregivers in China. Transplant Proc. 2017;49(9):1975–81. doi: 10.1016/j.transproceed.2017.09.022 29149947

[10] Zeng M, Li H, Song X, Jiang J, Chen Y. Factors Associated with Willingness toward Organ Donation in China: A Nationwide Cross-Sectional Analysis Using a Social-Ecological Framework. Healthcare (Basel). 2023;11(6):824. doi: 10.3390/healthcare11060824 36981481 PMC10048496

[11] Rochelle TL, Ng JS. Examining behavioural intention towards organ donation in Hong Kong. J Health Psychol. 2023;28(1):17–29. doi: 10.1177/13591053221092857 35443819

[12] Bandura A. Self-efficacy: toward a unifying theory of behavioral change. Psychol Rev. 1977;84(2):191–215. doi: 10.1037//0033-295x.84.2.191 847061

[13] Symvoulakis E, Markaki A, Rachiotis G, Linardakis M, Klinis S, Morgan M. Organ donation attitudes and general self-efficacy: exploratory views from a rural primary care setting. Rural Remote Health. 2019;19(4):5241. doi: 10.22605/RRH5241 31661290

[14] Wu AMS, Tang CS, Yogo M. Death anxiety, altruism, self‐efficacy, and organ donation intention among Japanese college students: A moderated mediation analysis. Australian Journal of Psychology. 2013;65(2):115–23. doi: 10.1111/ajpy.12003

[15] Bresnahan M, Lee SY, Smith SW, Shearman S, Nebashi R, Park CY, et al. A theory of planned behavior study of college students’ intention to register as organ donors in Japan, Korea, and the United States. Health Commun. 2007;21(3):201–11. doi: 10.1080/10410230701307436 17567252

[16] Figueroa CA, Mesfum ET, Acton NT, Kunst AE. Medical students’ knowledge and attitudes toward organ donation: results of a Dutch survey. Transplant Proc. 2013;45(6):2093–7. doi: 10.1016/j.transproceed.2013.02.135 23953518

[17] Wale J, Arthur A, Faull C. An analysis of knowledge and attitudes of hospice staff towards organ and tissue donation. BMJ Support Palliat Care. 2014;4(1):98–103. doi: 10.1136/bmjspcare-2012-000416 24644781

[18] Chung J, Choi D, Park Y. Knowledge and Opinions of Deceased Organ Donation Among Middle and High School Students in Korea. Transplant Proc. 2015;47(10):2805–9. doi: 10.1016/j.transproceed.2015.09.057 26707292

[19] Marck CH, Weiland TJ, Neate SL, Hickey BB, Jelinek GA. Australian emergency doctors’ and nurses’ acceptance and knowledge regarding brain death: a national survey. Clin Transplant. 2012;26(3):E254–60. doi: 10.1111/j.1399-0012.2012.01659.x 22583165

[20] Aijing L, Wenzhao X, Wei W, Qiquan W, Xuantong D. Public Opinion on Organ Donation After Death and Its Influence on Attitudes Toward Organ Donation. Ann Transplant. 2016;21:516–24. doi: 10.12659/aot.899268 27535587

[21] Lei L, Deng J, Zhang H, Dong H, Luo Y, Luo Y. Level of Organ Donation-Related Knowledge and Attitude and Willingness Toward Organ Donation Among a Group of University Students in Western China. Transplant Proc. 2018;50(10):2924–31. doi: 10.1016/j.transproceed.2018.02.095 30577149

[22] Siminoff LA, Chansiri K, Alolod G, Gardiner HM. Culturally Tailored and Community-Based Social Media Intervention to Promote Organ Donation Awareness among Asian Americans: “Heart of Gold”. J Health Commun. 2022;27(7):450–9. doi: 10.1080/10810730.2022.2119445 36062983 PMC10576892

[23] Khalaila R. Religion, altruism, knowledge and attitudes toward organ donation: a survey among a sample of Israeli college students. Med Law. 2013;32(1):115–29. 23781768

[24] Milaniak I, Wilczek-Rużyczka E, Przybyłowski P. Role of Empathy and Altruism in Organ Donation Decisionmaking Among Nursing and Paramedic Students. Transplant Proc. 2018;50(7):1928–32. doi: 10.1016/j.transproceed.2018.02.153 30177082

[25] Bolt S, Eisinga R, Venbrux E, Kuks JBM, Gerrits PO. Personality and motivation for body donation. Ann Anat. 2011;193(2):112–7. doi: 10.1016/j.aanat.2011.01.005 21330124

[26] Government HK. Yearbook - Religion. Hong Kong Government. 2010. https://web.archive.org/web/20180708031704/ http://www.yearbook.gov.hk/2010/en/pdf/C15.pdf

[27] Cai Y. On the impacts of traditional Chinese culture on organ donation. J Med Philos. 2013;38(2):149–59. doi: 10.1093/jmp/jht007 23449366

[28] Doerry K, Oh J, Vincent D, Fischer L, Schulz-Jürgensen S. Religious and cultural aspects of organ donation: Narrowing the gap through understanding different religious beliefs. Pediatr Transplant. 2022;26(7):e14339. doi: 10.1111/petr.14339 35735257

[29] Pyszczynski T, Greenberg J, Solomon S. A dual-process model of defense against conscious and unconscious death-related thoughts: an extension of terror management theory. Psychol Rev. 1999;106(4):835–45. doi: 10.1037/0033-295x.106.4.835 10560330

[30] Moonajilin MS, Banik R, Islam MS, Ishadi KS, Hosen I, Gesesew HA, et al. Understanding the Public’s Viewpoint on Organ Donation Among Adults in Bangladesh: An Exploratory Cross-Sectional Study. Health Sci Rep. 2024;7(12):e70254. doi: 10.1002/hsr2.70254 39698525 PMC11652383

[31] Wu AMS, Lu L. Cognitive obstacles against organ donation: The influence of negative attitudes, norms, and traditional beliefs on Chinese people’s intention to donate organs after death. Community & Applied Soc Psy. 2010;21(1):87–93. doi: 10.1002/casp.1054

[32] Kogut T, Pittarello A, Slovic P. The Fear of Personal Death and the Willingness to Commit to Organ Donation. Social Psychological and Personality Science. 2023;15(6):630–8. doi: 10.1177/19485506231198135

[33] Bayrak M, Düzova ÜS. Does Death Anxiety Affect the Attitude Towards Organ Donation? Transplant Proc. 2023;55(2):263–7. doi: 10.1016/j.transproceed.2023.02.017 36918313

[34] Nolan BE, McGrath PJ. Social-cognitive influences on the willingness to donate organs. Organ donation and transplantation: Psychological and behavioral factors. Washington, DC, US: American Psychological Association. 1992:25–36.

[35] Jessop DC, Wade J. Fear appeals and binge drinking: a terror management theory perspective. Br J Health Psychol. 2008;13(Pt 4):773–88. doi: 10.1348/135910707X272790 18208639

[36] Hollon SD, Kendall PC. Cognitive self-statements in depression: Development of an automatic thoughts questionnaire. Cogn Ther Res. 1980;4(4):383–95. doi: 10.1007/bf01178214

[37] Kupferberg A, Bicks L, Hasler G. Social functioning in major depressive disorder. Neurosci Biobehav Rev. 2016;69:313–32. doi: 10.1016/j.neubiorev.2016.07.002 27395342

[38] Xin Z, Li S, Jia Y, Yuan H. Analysis of self-healing of depression by helping others in adolescents from the perspective of constructivism. Front Psychiatry. 2023;14:1201923. doi: 10.3389/fpsyt.2023.1201923 37599875 PMC10437064

[39] O’Connor LE, Berry JW, Lewis T, Mulherin K, Crisostomo PS. Empathy and depression: The moral system on overdrive. Empathy in mental illness. New York, NY, US: Cambridge University Press. 2007:49–75.

[40] 我好像救过我自己. http://xhslink.com/a/2lX3O2fEwzxdb. 2024.

[41] Xiong X, Lai K, Jiang W, Sun X, Dong J, Yao Z, et al. Understanding public opinion regarding organ donation in China: A social media content analysis. Sci Prog. 2021;104(2). doi: 10.1177/00368504211009665 33861661 PMC10454996

[42] Ajzen I. The theory of planned behavior. Organizational Behavior and Human Decision Processes. 1991;50(2):179–211. doi: 10.1016/0749-5978(91)90020-t

[43] Fishbein M, Ajzen I. Belief, attitude, intention and behaviour: An introduction to theory and research. 1975.

[44] Stephenson MT, Morgan SE, Roberts-Perez SD, Harrison T, Afifi W, Long SD. The role of religiosity, religious norms, subjective norms, and bodily integrity in signing an organ donor card. Health Commun. 2008;23(5):436–47. doi: 10.1080/10410230802342119 18850391

[45] Schmitt DP, Long AE, McPhearson A, O’Brien K, Remmert B, Shah SH. Personality and gender differences in global perspective. Int J Psychol. 2017;52 Suppl 1:45–56. doi: 10.1002/ijop.12265 27000535

[46] Zhang X, Zheng X, Chen T, Li Y, Wang Y, Chen J, et al. Factors affecting acceptance of organ donation in mainland China: A national cross-sectional study. J Clin Nurs. 2023;32(15–16):5219–29. doi: 10.1111/jocn.16587 36448208

[47] Wong SH, Chow AYM. Preliminary Development of a Postmortem Bodily Integrity Concerns Scale Among the University Students in Hong Kong. Omega (Westport). 2021;84(2):617–33. doi: 10.1177/0030222820904887 32108544

[48] Wall ER. Regression and Effect Size. Journal of Visual Impairment & Blindness. 2023;117(2):191–2.

[49] Wang F, Wu Y, Sun X, Wang D, Ming W-K, Sun X, et al. Reliability and validity of the Chinese version of a short form of the family health scale. BMC Prim Care. 2022;23(1):108. doi: 10.1186/s12875-022-01702-1 35524178 PMC9077878

[50] Carciofo R, Yang J, Song N, Du F, Zhang K. Psychometric Evaluation of Chinese-Language 44-Item and 10-Item Big Five Personality Inventories, Including Correlations with Chronotype, Mindfulness and Mind Wandering. PLoS One. 2016;11(2):e0149963. doi: 10.1371/journal.pone.0149963 26918618 PMC4769279

[51] Cao R, Cheng S, Tang W, Song H. The Reliability and Validity of Automatic Thoughts Questionnaire. Chinese J clinical psychology. 2001;2.

[52] Weber K, Martin MM, Corrigan M. Real Donors, Real Consent: Testing the Theory of Reasoned Action on Organ Donor Consent. J Applied Social Pyschol. 2007;37(10):2435–50. doi: 10.1111/j.1559-1816.2007.00265.x

[53] Demir B, Kumkale GT. Individual differences in willingness to become an organ donor: A decision tree approach to reasoned action. Personality and Individual Differences. 2013;55(1):63–9. doi: 10.1016/j.paid.2013.02.002

[54] Fan X, Li M, Rolker H, Li Y, Du J, Wang D, et al. Knowledge, attitudes and willingness to organ donation among the general public: a cross-sectional survey in China. BMC Public Health. 2022;22(1):918. doi: 10.1186/s12889-022-13173-1 35534843 PMC9082919

[55] Gong F, Jia Y, Zhang J, Cao M, Jia X, Sun X, et al. Media use and organ donation willingness: A latent profile analysis from Chinese residents. Front Public Health. 2022;10:1000158. doi: 10.3389/fpubh.2022.1000158 36324440 PMC9618944

[56] John OP, Donahue EM, Kentle RL. The Big Five Inventory---versions 4a and 5. 1991.

[57] John OP, Naumann LP, Soto CJ. Paradigm shift to the integrative Big Five trait taxonomy: History, measurement, and conceptual issues. Handbook of personality: Theory and research. 3rd ed. New York, NY, US: The Guilford Press. 2008:114–58.

[58] Brug J, Vugt MV, van Den Borne B, Brouwers A, Hooff HV. Predictors of willingness to register as an organ donor among dutch adolescents. Psychology & Health. 2000;15(3):357–68. doi: 10.1080/08870440008401998

[59] Lam WA, McCullough LB. Influence of religious and spiritual values on the willingness of Chinese-Americans to donate organs for transplantation. Clin Transplant. 2000;14(5):449–56. doi: 10.1034/j.1399-0012.2000.140502.x 11048989

[60] White CJM, Norenzayan A, Schaller M. The Content and Correlates of Belief in Karma Across Cultures. Pers Soc Psychol Bull. 2019;45(8):1184–201. doi: 10.1177/0146167218808502 30554555

[61] Morgan S, Miller J. Communicating about gifts of life: the effect of knowledge, attitudes, and altruism on behavior and behavioral intentions regarding organ donation. Journal of Applied Communication Research. 2002;30(2):163–78. doi: 10.1080/00909880216580

[62] Kopfman JE, Smith SW. Understanding the audiences of a health communication campaign: A discriminant analysis of potential organ donors based on intent to donate. Journal of Applied Communication Research. 1996;24(1):33–49. doi: 10.1080/00909889609365438

[63] Hayes AF. Introduction to mediation, moderation, and conditional process analysis: A regression-based approach. New York, NY, US: Guilford Press. 2013.

[64] Morgan SE, Stephenson MT, Harrison TR, Afifi WA, Long SD. Facts versus “Feelings”: how rational is the decision to become an organ donor? J Health Psychol. 2008;13(5):644–58. doi: 10.1177/1359105308090936 18519438

[65] Besser A, Amir M, Barkan S. Who signs an organ transplant donor card? A study of personality and individual differences in a sample of Israeli university students. Personality and Individual Differences. 2004;36(7):1709–23. doi: 10.1016/j.paid.2003.07.012

[66] Cheng B, Ho C-P, Ho S, Wong A. An Overview on Attitudes Towards Organ Donation in Hong Kong. Hong Kong Journal of Nephrology. 2005;7(2):77–81. doi: 10.1016/s1561-5413(09)60218-0

[67] Chung CKY, Ng CWK, Li JYC, Sum KCY, Man AHY, Chan SPC, et al. Attitudes, knowledge, and actions with regard to organ donation among Hong Kong medical students. Hong Kong Med J. 2008;14(4):278–85. 18685160

[68] Wong SH, Chow AYM. Preliminary Development of a Postmortem Bodily Integrity Concerns Scale Among the University Students in Hong Kong. Omega (Westport). 2021;84(2):617–33. doi: 10.1177/0030222820904887 32108544

[69] Braun KL, Nichols R. Death and dying in four Asian American cultures: a descriptive study. Death Stud. 1997;21(4):327–59. doi: 10.1080/074811897201877 10170477

[70] Gong X, Zhang F, Fung HH. Are Older Adults More Willing to Donate? The Roles of Donation Form and Social Relationship. J Gerontol B Psychol Sci Soc Sci. 2019;74(3):440–8. doi: 10.1093/geronb/gbx099 28977660

[71] Lai Y-C, Lee W-C, Juang Y-Y, Yen L-L, Weng L-C, Chou HF. Effect of social support and donation-related concerns on ambivalence of living liver donor candidates. Liver Transpl. 2014;20(11):1365–71. doi: 10.1002/lt.23952 25044400

[72] Sanner MA. People’s attitudes and reactions to organ donation. Mortality. 2006;11(2):133–50. doi: 10.1080/13576270600615351

[73] Moorlock G, Ives J, Draper H. Altruism in organ donation: an unnecessary requirement?. J Med Ethics. 2014;40(2):134–8. doi: 10.1136/medethics-2012-100528 23538329 PMC3913211

[74] Dopelt K, Siton L, Harrison T, Davidovitch N. Revisiting the Relationship between Altruism and Organ Donation: Insights from Israel. Int J Environ Res Public Health. 2022;19(12):7404. doi: 10.3390/ijerph19127404 35742655 PMC9223858

[75] Hill EM. Posthumous organ donation attitudes, intentions to donate, and organ donor status: Examining the role of the big five personality dimensions and altruism. Personality and Individual Differences. 2016;88:182–6. doi: 10.1016/j.paid.2015.09.021

[76] Tomer A, Eliason G. Attitudes about life and death: Toward a comprehensive model of death anxiety. Death attitudes and the older adult: Theories, concepts, and applications. New York, NY, US: Brunner-Routledge. 2000:3–22.

[77] Blackie LER, Cozzolino PJ. Of blood and death: a test of dual-existential systems in the context of prosocial intentions. Psychol Sci. 2011;22(8):998–1000. doi: 10.1177/0956797611415542 21742931

[78] Zeng T, Tse C-S. Does the mortality salience effect on worldview defence depend on the cultural orientation of Chinese people? Int J Psychol. 2020;55(2):291–304. doi: 10.1002/ijop.12562 30592038

[79] Xiao Q, He W, Zhu Y. Re-examining the relationship between mortality salience and prosocial behavior in Chinese context. Death Stud. 2017;41(4):251–5. doi: 10.1080/07481187.2016.1268220 27918862

[80] Hirschberger G, Ein-Dor T, Almakias S. The self-protective altruist: terror management and the ambivalent nature of prosocial behavior. Pers Soc Psychol Bull. 2008;34(5):666–78. doi: 10.1177/0146167207313933 18303130

[81] Fry PS. Perceived self-efficacy domains as predictors of fear of the unknown and fear of dying among older adults. Psychol Aging. 2003;18(3):474–86. doi: 10.1037/0882-7974.18.3.474 14518809

[82] Feeley TH. College Students’ Knowledge, Attitudes, and Behaviors Regarding Organ Donation: An Integrated Review of the Literature1. J Applied Social Pyschol. 2007;37(2):243–71. doi: 10.1111/j.0021-9029.2007.00159.x

