# Supplementary material for: Why don’t they want to donate? Cultural and psychological factors influencing the organ donation intention among Hong Kong University students
Source: PLoS One. 2025 Dec 19;20(12):e0338201. doi: 10.1371/journal.pone.0338201 (PMC12716731; doi:10.1371/journal.pone.0338201)

Appendix A

Fig 1. Hypothetical mediation model of depressive thinking and organ donation intention

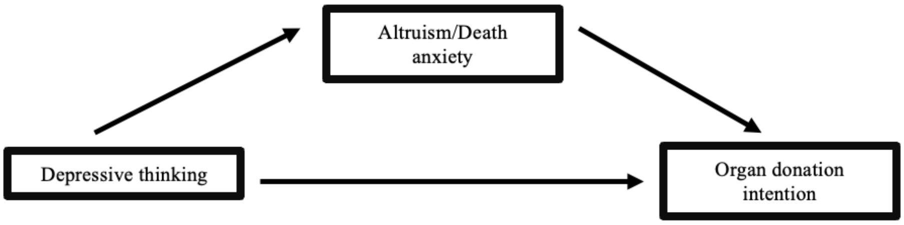

Fig 2. Hypothetical moderation model of self-efficacy and organ donation intention

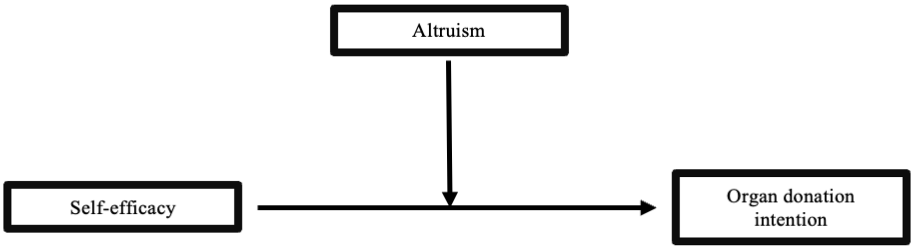

Fig 3. Hypothetical moderated mediation model of self-efficacy and organ donation intention

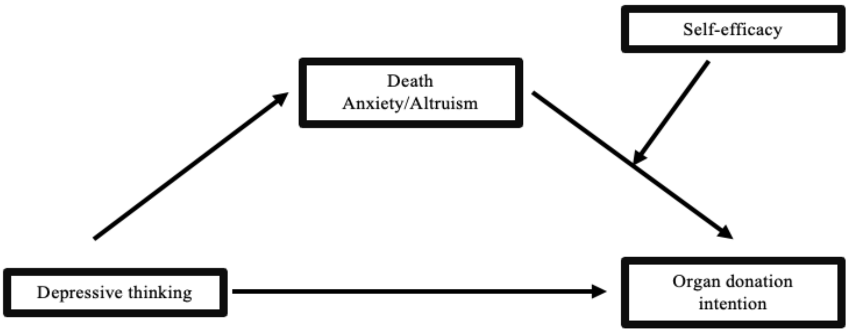

Supplement: S1 Appendix — (PDF) [file pone.0338201.s001.pdf]
