# Supplementary material for: Why don’t they want to donate? Cultural and psychological factors influencing the organ donation intention among Hong Kong University students
Source: PLoS One. 2025 Dec 19;20(12):e0338201. doi: 10.1371/journal.pone.0338201 (PMC12716731; doi:10.1371/journal.pone.0338201)
Supplement: S2 Appendix — (PDF) [file pone.0338201.s002.pdf]

Appendix B

Organ donation intentions questionnaire

器官捐贈意向問卷

Session 1: Demographic Information

Age您的年齡:

Gender 您的性別:

Education Level 所在年級:

- Year 1 大一
- Year 2 大二
- Year 3 大三
- Year 4 大四
- Year 5 or above 大五（或以上）

Family household income 家庭每月收入:

- <10,000 HKD港幣
- 10,000 – 29,999 HKD港幣
- 30,000 – 49,999 HKD港幣
- 50,000 – 69,999 HKD港幣
- 70,000 – 89,999 HKD港幣

- >=90,000 HKD港幣

Father’s education level 父親教育水平:

- Lower secondary or below 中學（初中）及以下
- Higher secondary / diploma 中學/專業文憑
- Bachelor’s degree本科
- Postgraduate degree or above 碩士及以上

Mother’s education level 母親教育水平:

- Lower secondary or below 中學（初中）及以下
- Higher secondary / diploma 中學/專業文憑
- Bachelor’s degree本科
- Postgraduate degree or above 碩士及以上

Your own monthly income 個人每月收入:

- <1,000 HKD港幣
- 1,000 – 3,999 HKD港幣
- 4,000 – 5,999 HKD港幣
- 6,000 – 7,999 HKD港幣
- 8,000 – 9,999 HKD港幣
- >=10,000 HKD港幣

Have you (ever) clinically diagnosed as any type of depression or depressive disorder (including Major Depressive Disorder, Chronic Depression. Depression in Bipolar Disorder, Seasonal Depression, Pregnancy-Related Depression, Psychotic Depression, PMS Depression, Non-Typical Depression, Melancholic Depression)? 您是否（曾經）被臨床診斷為患有任何類型的抑鬱症或抑鬱障礙（包括但不限於重度抑鬱症、慢性抑鬱症、雙相情感障礙中的抑鬱症、季節性抑鬱症、孕期相關抑鬱症、精神病性抑鬱症、經前抑鬱症、非典型抑鬱症和憂鬱型抑鬱症）？

- Yes 是
- No 否

Session 2: Organ Donation intentions and Registration Status

Are you willing to donate organs after death? 您是否願意在死後捐獻器官?

- Yes 願意
- No 不願意

Have you filled in the organ donation registration form? 您是否填寫過捐獻器官異體的報名登記表?

- Yes 是
- No 否

Session 3: Psychological and Emotional Factors of Organ donation

*Items with “(R)” are reverse-scored. (This will not be shown to the participants.)

***Knowledge of organ donation* (T/F)** 器官捐贈知識（是/否）

1. Organ donation refers to donation of cadaveric organs, living organs cannot be donated (F)

器官捐贈是指捐贈屍體器官，活體器官不能捐贈。（否）

2. Brain death means that the patient cannot breathe, and the heart cannot beat (F)

腦死亡意味著病人無法呼吸，心臟無法跳動。（否）

3. Living organs can only be donated to immediate family members (F)

活體器官只能捐贈給直系親屬。（否）

4. Any doctor can determine brain death (F)

任何醫生都可以判斷腦死亡。（否）

5. Organ removal must be performed only after brain death is determined (T)

器官摘除必須在確定腦死亡後進行。（是）

6. People with any disease can donate organs (F)

患有任何疾病的人都可以捐贈器官。（否）

7. People of any age can donate organs (T)

任何年齡的人都可以捐贈器官。（是）

8. Citizens have not expressed their disapproval of organ donation during their lifetime. After their death, spouses, adult children, and parents can jointly express their consent to organ donation (T)

如果公民生前沒有表示反對器官捐贈，在其去世後，配偶、成年子女和父母可以共同表

示同意捐贈器官。（是）

9. Organ donors cannot claim any monetary compensation (T)

器官捐贈者不能要求任何金錢補償。（是）

10. Donors pay for organ removal surgery (F)

捐贈者需要支付器官摘除手術費用。（否）

Social Atmosphere 社會氛圍

1 = strongly disagree; 2 = slightly disagree; 3 = Neither agree nor disagree; 4 = slightly agree; 5 = strongly agree

1 = 非常不同意; 2 = 有點不同意; 3 = 既不同意也不反對; 4 = 有點同意; 5 = 非常同意

Social interaction/support 社會互動/支持:

1. My family is supportive of organ donation

我的家人支持器官捐贈。

2. My friends are supportive of organ donation

我的朋友支持器官捐贈。

3. If asked by their son or daughter, parents are likely to sign up to become an organ donor

如果孩子提出要求，父母很可能會登記成為器官捐贈者。

4. If asked by a friend, a person is likely to sign up to become an organ donor

如果朋友提出請求，人們很可能會登記成為器官捐贈者。

5. I have had discussions with others about being an organ donor

我曾經與他人討論過成為器官捐贈者的話題。

6. My fellow university students are supportive of organ donation

我的大學同學支持器官捐贈。

*Social media use*社交媒體運用:

1. Choose one social media that use most: 1. Newspaper 2. Magazines 3. Broadcast 4. TV 5. Books (non-textbook) 6. Personal computers (including tablets) 7. Smartphones

請選擇您最常使用的社交媒體類型: 1. 報紙, 2. 雜誌, 3. 廣播, 4. 電視, 5. 圖書（非教材）, 6. 個人電腦（包括平板電腦）, 7. 智能手機

2. How frequently you use social media? (Never use =0; Occasionally use=1; Sometimes use =2; Often use =3; Almost daily= 4.)

您使用社交媒體的頻率是多少? （從不使用= 0;偶爾使用= 1;有時使用= 2;經常使用= 3;幾乎每天使用= 4）

3. Indicate the number of days you use social media in a week. (0-7)

請指出您每周使用社交媒體的天數。（0-7 天）

Personality Traits 性格特質

Please make your choice in relation to your situation. Questions are positively scored: 1 = strongly disagree; 2 = slightly disagree; 3 = Neither agree nor disagree; 4 = slightly agree; 5 = strongly agree

1 = 非常不同意; 2 = 有點不同意; 3 = 既不同意也不反對; 4 = 有點同意; 5 = 非常同意

I see Myself as Someone Who...

1. Talks a lot 愛說話，健談

2. Notices other people’s weak points 常常挑別人的毛病

3. Does things carefully and completely 做事仔細且徹底，有始有終

4. Is sad, depressed 感到憂鬱或沮喪

5. Is original, comes up with new ideas 有創意，能想出新點子

6. Keeps their thoughts to themselves 把想法藏在心裡

7. Is helpful and not selfish with others 樂於助人，不自私

8. Can be kind of careless 有時會有點粗心大意

9. Is relaxed, handles stress well 很放鬆，而且能很好地應對壓力

10. Is curious about lots of different things 對很多不同的事情感到好奇

11. Has a lot of energy 充滿了活力

12. Starts arguments with others 會挑起和別人的爭執

13. Is a good, hard worker 是一個努力工作的人

14. Can be tense; not always easy going 有時會緊張，不總是那麼隨和

15. Clever; thinks a lot 很聰明，會思考很多

16. Makes things exciting 讓事情變得有趣

17. Forgives others easily 生性寬容

18. Isn’t very organized 不是很有條理

19. Worries a lot 總是擔心很多事情

20. Has a good, active imagination 有豐富的想像力

21. Tends to be quiet 往往很安靜

22. Usually trusts people 通常是相信別人的

23. Tends to be lazy 有時會偷懶

24. Doesn’t get upset easily; steady不容易被激怒，個性穩重

25. Is creative and inventive 有創意，善於發明

26. Has a good, strong personality 我有堅定而自信的性格

27. Can be cold and distant with others 有時對人冷漠，漠不關心

28. Keeps working until things are done 會堅持工作直到完成

29. Can be moody 情緒多變，喜怒無常

30. Likes artistic and creative experiences 喜歡藝術和創意的體驗

31. Is kind of shy 有時會害羞或拘謹

32. Kind and considerate to almost everyone 對幾乎所有人都親切和體貼

33. Does things quickly and carefully 做事迅速且仔細

34. Stays calm in difficult situations 在緊張的情況下能保持冷靜

35. Likes work that is the same every time 喜歡每次都一樣的工作內容

36. Is outgoing; likes to be with people 性格外向，喜歡和人相處

37. Is sometimes rude to others 有時對人很粗魯

38. Makes plans and sticks to them 會制定計劃並堅持執行

39. Get nervous easily 容易緊張

40. Likes to think and play with ideas 喜歡思考，想法機靈

41. Doesn’t like artistic things (plays, music) 不喜歡藝術類的東西（如戲劇、音樂）

42. Likes to cooperate; goes along with others 喜歡合作，願意配合別人

43. Has trouble paying attention 很難集中注意力

44. Knows a lot about art, music, and books 對藝術、音樂和書籍瞭解很多

Self-efficacy 自我效能

1 = strongly disagree; 2 = slightly disagree; 3 = neither agree nor disagree; 4 = slightly agree; 5 = strongly agree

1 = 非常不同意; 2 = 有點不同意; 3 = 既不同意也不反對; 4 = 有點同意; 5 = 非常同意

1. It is difficult to register as an organ donor by signing an organ donor card.

簽署器官捐贈卡成為一名器官捐贈者很困難。

2. Signing an organ donor card is too much trouble.

簽署器官捐贈卡太麻煩了。

3. The decision to sign an organ donor card is difficult.

決定簽署器官捐贈卡太難了。

Family Dynamics 家庭健康

Based on your personal beliefs, please rate how much you agree or disagree with each of the following statements. 請根據您的個人想法，評估您對以下每一項陳述的同意或不同意程度。

1 = strongly disagree; 2 = slightly disagree; 3 = neither agree nor disagree; 4 = slightly agree; 5 = strongly agree

1 = 非常不同意; 2 = 有點不同意; 3 = 既不同意也不反對; 4 = 有點同意; 5 = 非常同意

1. We support each other.

我們互相支持。

2. I feel safe in my family relationships.

我在家庭關係中感到有安全感。

3. We help each other in seeking health care services when needed (such as making doctor's appointments).

在需要的時候，我們彼此尋求醫療服務（例如掛號）。

4. We help each other in making healthy changes.

我們互相幫助為了健康而做出改變。

5. We are full of hope even in difficult times.

在很艱難的時候，我們也保持希望。

6. We do not trust doctors or other health professionals (R).

我們不信任醫護人員。

7. When we have problems at work or school, we can turn to people other than our family for help.

當我們在學校或工作中遇到問題時，我們可以向家人以外的人尋求協助。

8. My family did not have enough money at the end of the month after bills were paid (R).

在負擔基本的生活開銷後，我們家就沒有閒錢了。

9. My family did not have adequate housing (R).

我家的住房不能滿足家庭的需求。

Beliefs in Karma/Buddhism 因果報應/佛教信仰

Based on your personal beliefs, please rate how much you agree or disagree with each of the following statements. 請根據您的個人想法，評估您對以下每一項陳述的同意或不同意程度。

1 = strongly disagree; 2 = slightly disagree; 3 = neither agree nor disagree; 4 = slightly agree; 5 = strongly agree

1 = 非常不同意; 2 = 有點不同意; 3 = 既不同意也不反對; 4 = 有點同意; 5 = 非常同意

Buddhist beliefs toward organ donation 佛教對器官捐贈的觀念*:*

1. Taking organs from a person who has just died will still cause him great pain and disturb his dying process before he is born again. 從剛剛去世的人身上取器官，會使他感受到極大的痛苦，並干擾他臨終的過程，影響他來世的轉世。

2. A person who wishes to donate his organs after his death is being very compassionate, and this will help give him a better rebirth. 願意在死後捐贈器官的人展現了極大的慈悲心，這將有助於他在來世獲得更好的轉世。

3. Taking organs from a person who has just died will cause him to have a bad rebirth. 從剛去世的人身上取器官，會導致他在來世遭遇不良的轉世。

Belief in Karma 因果報應*:*

1. Karma is a force that influences the events that happen in my life.

因果報應是一種影響我生活事件的力量。

2. Karma is not something real (R)

因果報應不是真實的事物。

3. Karma is a force that influences the events that happen in other people’s lives

因果報應是一種影響其他人生活事件的力量。

4. When people are met with misfortune, they have brought it upon themselves by previous behavior in their life

當人們遇到不幸時，他們是因為之前的行為而自作自受。

5. When people experience good fortune, they have brought it upon themselves by previous behavior in their life

當人們經歷幸運時，他們是因為之前的行為而獲得的。

6. If a person does something bad, even if there are no immediate consequences, they will be punished for it in some future time in their life

如果一個人做了壞事，即使沒有立即後果，他們也會在未來某個時間受到懲罰。

7. When someone does a good deed, even if there are no immediate consequences, they will be rewarded for it in some future time in their life

當某人做好事時，即使沒有立即後果，他們也會在未來某個時間受到獎勵。

8. In the long-run, good things happen to good people and bad things happen to bad people

從長遠來看，好人會有好報，壞人會有惡報。

9. When people are met with misfortune, they have brought it upon themselves by behavior in a past life

當人們遇到不幸時，他們是因為前世的行為而自作自受。

10. When people experience good fortune, they have brought it upon themselves by behavior in a past life

當人們經歷幸運時，他們是因為前世的行為而獲得的。

11. If a person does something bad, even if there are no immediate consequences, they will be punished for it in a future life

如果一個人做了壞事，即使沒有立即後果，他們也會在來世受到懲罰。

12. When someone does a good deed, even if there are no immediate consequences, they will be rewarded for it in a future life

當某人做好事時，即使沒有立即後果，他們也會在來世受到獎勵。

13. After people die, they are reborn in a new body

當人們去世後，他們會以新身體轉世重生。

14. There is no such thing as rebirth or reincarnation (R)

沒有輪迴或轉世這回事。

15. People’s moral behavior during their current life influences their rebirth in a future life

人們在現世的道德行為會影響他們來世的輪迴。

16. The ultimate goal of life is freedom from the cycle of birth and death

生命的最終目標是脫離生死輪迴。

Altruism 利他行為

Based on your personal beliefs, please rate how much you agree or disagree with each of the following statements. 請根據您的個人想法，評估您對以下每一項陳述的同意或不同意程度。

1 = strongly disagree; 2 = slightly disagree; 3 = neither agree nor disagree; 4 = slightly agree;

5 = strongly agree

1 = 非常不同意; 2 = 有點不同意; 3 = 既不同意也不反對; 4 = 有點同意; 5 = 非常同意

1. Overall, I tend to be a cheerful person.

總的來說，我傾向於是一個快樂的人。

2. I am not what I would call a warm-hearted person. (R)

我不是一個可以被稱為熱心的人。

3. When people hurt me, I usually hold a grudge for a long time. (R)

當別人傷害我時，我通常會記仇很久。

4. I am an affectionate and tender person.

我是一個有愛心且溫柔的人。

5. I am generally a sincere and truthful person.

我通常是一個真誠且誠實的人。

6. If I could help save somebody's life, I would do everything possible.

如果我可以幫助拯救某人的生命，我會竭盡全力去做。

7. I enjoy doing small favors every day for the people I care about.

我喜歡每天為我關心的人幫一些小忙。

8. Helping others is one of the most important aspects of life.

幫助他人是生活中最重要的事情之一。

9. I enjoy working for the welfare of others.

我享受為他人的福祉而努力的過程。

10. My family tends to do what we can to help those less fortunate than ourselves. 28

我的家庭往往會盡其所能幫助那些不如我們幸運的人。

11. I agree with the old saying, “It is better to give than to receive”.

我同意這句老話:“施比受更有福”。

Depressive Thinking 負面想法

Please read each thought and indicate how frequently, if at all, the thought has occurred to you over the past week. Please read each item carefully and circle the appropriate answers on the answer sheet in the following fashion:

1 = not at all; 2 = sometimes; 3 = moderately often; 4 = often; 5 = all the time

請閱讀以下每個想法，並指出在過去一週內這些想法出現的頻率。請仔細閱讀每一項，並在答案表上圈出適當的答案，按以下方式評分：1 = 完全沒有；2 = 有時候；3 = 偶爾；4 = 經常；5 = 一直如此

1. I feel like I’m up against the world. 我覺得活在世上困難重重。

2. I’m no good. 我不好。

3. Why can’t I ever succeed? 為什麼我總不能成功?

4. No one understands me. 沒有人理解我。

5. I’ve let people down. 我讓人失望。

6. I don’t think I can go on. 我覺得過不下去了。

7. I wish I were a better person. 真希望我能好一點。

8. I’m so weak. 我很虛弱。

9. My life is not going the way I want it to. 我的生活不按我的願望發展。

10. I’m so disappointed in myself. 我對自己很不滿意。

11. Nothing feels good anymore. 我覺得一切都不好了。

12. I can’t understand this anymore. 我無法堅持下去。

13. I can’t get started. 我無法重新開始。

14. What’s wrong with me? 我究竟犯了什麼毛病？

15. I wish I were somewhere else. 真希望我是在另外一個地方。

16. I can’t get things together. 我無法同時應付這些事情。

17. I hate myself. 我恨我自己。

18. I’m worthless. 我毫無價值。

19. I wish I could just disappear. 真希望我一下子就消失了。

20. What’s the matter with me? 我這是怎麼了？

21. I’m a loser. 我是個失敗者。

22. My life is a mess. 我的生活一團糟。

23. I’m a failure. 我一事無成。

24. I’ll never make it. 我不可能幹好。

25. I feel so helpless. 我覺得孤立無援。

26. Something has to change. 有些東西必須改變。

27. There must be something wrong with me. 我肯定有問題。

28. My future is bleak. 我的將來毫無希望。

29. It’s just not worth it. 這根本毫無價值。

30. I can’t finish anything. 我幹什麼事都有頭無尾。

Death Anxiety 死亡焦慮

1 = strongly disagree; 2 = slightly disagree; 3 = neither agree nor disagree; 4 = slightly agree; 5 = strongly agree

1 = 非常不同意; 2 = 有點不同意; 3 = 既不同意也不反對; 4 = 有點同意; 5 = 非常同意

1. I get upset when I am in a cemetery

當我在墓地時，我會感到不安。

2. The certainty of death makes life meaningless

死亡的必然性讓生命變得毫無意義。

3. It annoys me to hear about death

聽到有關死亡的話題讓我心煩。

4. I find it difficult to accept the idea that it all finishes with death

我很難接受死亡就是一切結束這個想法。

5. I think I would be happier if I ignored the fact that I have to die

我認為如果我忽視自己必須死亡這個事實，我會更快樂。

6. I think I am more afraid of death than most people

我覺得自己比大多數人更害怕死亡。

7. I find it really difficult to accept that I have to die

我覺得接受自己必須死亡這件事非常困難。

8. I would never accept a job in a funeral home

我絕不會接受一份在殯儀館工作的職位。

9. The idea that there is nothing after death frightens me

死後什麼都沒有的想法讓我感到害怕。

10. The idea of death troubles me.

死亡的想法讓我感到困擾。

11. I very often think about the cause of my death.

我經常想自己會因為什麼原因而死亡。

12. Coffins makes me nervous

棺材讓我感到緊張。

13. I am worried about what's after death

我擔心死後的事情。

14. I often think I may have a serious disease

我常常擔心自己可能患有嚴重疾病。

15. Dying is the worst thing that could happen to me

死亡是我能想到最糟糕的事情。

16. The sight of a corpse deeply shocks me

見到屍體的景象會讓我感到極度震驚。

17. I frequently think of my own death

我經常想到自己的死亡。
